# The Comparison of Chlorhexidine Solution and Swab With Toothbrush and Toothpaste Effect on Preventing Oral Lesions in Hospitalized Patients in Intensive Care Unit

**DOI:** 10.5539/gjhs.v8n5p211

**Published:** 2015-10-20

**Authors:** Zahra Estaji, Mohammad Alinejad, Mohammad Hassan Rakhshani, Mojtaba Rad

**Affiliations:** 1 Faculty of Nurse, Sabzevar University of Medical Sciences, Sabzevar, Iran; 2 Department of Biostatistics, Faculty of Health, Sabzevar University of Medical Sciences, Sabzevar, Iran

**Keywords:** intensive care, oral lesions, toothbrush, chlorhexidine

## Abstract

**Background:**

Maintaining of oral hygiene has been known as one of the basic tasks of nurses working at intensive care unit. This study aimed to compare the effectiveness of chlorhexidine solution with toothbrush in prevention of oral lesions or ulcers in the different parts of the mouth.

**Methods:**

In this clinical trial study, research Society included patients hospitalized with endotracheal tube since the arrival time in intensive care unit. In this study, 30 patients were selected with target-based approach and equally divided into two groups through the permutation blocking method for oral care toothbrush and toothpaste and using chlorhexidine and swab. The modified form of Beck Oral Assessment Scale (BOAS) and MPS were used to obtain needed information. Data were analyzed by means of R software (version 3.0.2) and also multiple logistic regressions in the confidence level of 95%.

**Results:**

This study indicated an association between using toothbrush and the oral health level (OR: 1.52). In different parts of the mouth, there was no difference between two groups in prevention of lesions in gums, lips and tongue while, this was significant in relation to plaque, mucus and teeth with an odd ratio of 3.94 for teeth and 2.75 for mucus. In comparison, there was an association between implying chlorhexidine and saliva health level. (OR: 2.046).

**Conclusion:**

This survey showed that using toothbrush has a noticeable impact on declining oral lesions in varied parts of the mouth.

## 1. Introduction

Oral care is one of the most important nursing activities and challenges in intensive care unit (Grap et al., 2003). The American association of critical care nurses illustrates that oral care programs must to be performed for maintaining moisture levels of mouth, lips and mucus to prevent damage of these parts (Ames, 2011). The first aim of oral care is to promote oral hygiene level which can lead to decreasing of oropharyngeal colonization and dental plaque produced by bacterial infections and also aspiration of saliva including this colony (Feider et al., 2011). Patients hospitalized in intensive care unit mostly suffer from a weak oral hygiene due to losing consciousness level and difficulties in swallowing. Among them, patients receiving mechanical ventilation are at higher risk since; intubation can result in an increased possibility of adherence of bacteria to the oral mucus (Ranjbar et al., 2011).

Ventilator associated pneumonia is one of the most common and dangerous hospital infections responsible for 20 -70 % of mortality rate and one-third of hospital infections in the intensive care unit. Some studies revealed a substantial association between oropharyngeal bacterial colonization and mechanical ventilation (Adib-Hajbaghery et al., 2011).

Oral clearance can cause a decrease in amount of dental plaque, periodontal diseases and ventilator associated pneumonia (Ames, 2011). In a healthy person, oral flora remains steady over the time whereas, 48 hours after hospitalization this flora is replaced by some negative microorganisms with a high potential of virulence and in case of suitable conditions, opportunistic pathogens will growth and cause some general and local complications.

Normally, fibronectin is present on cell surfaces and acts as a host defense mechanism, blocking pathogenic bacteria attachment to oral and tracheal mucus membranes. This depletion of fibronectin allows cell receptor sites to replace normal flora with virulent pathogens such as Pseudomonas aeruginosa on buccal and pharyngeal epithelial cells, a defensive material existing in the surface of mouth and teeth, drops in critically ill patients resulting in rising bacterial binding strength to the surface of teeth and oropharyngeal epithelial cells, in turn, causes developing of dental plaques (Adib-Hajbaghery et al., 2011).

Nursing students mostly were educated about oral care and in spite of its importance in caring of air passageways in patients receiving mechanical ventilation; there is a less attention to this (Sole et al., 2003). Some studies discus that oral care almost always not been performed based on a standard protocol in the intensive care unit; however, most patients suffer from oral complications due to some different diseases and medical measures. Patients hospitalized in the intensive care unit are further susceptible to develop dental plaque and other oral disorders (Ranjbar et al., 2010).

Oral care has been considered as a difficult procedure by 68% of nurses owing to lake of sufficient equipments and mechanical barriers in the mouth and it’s around (Grap et al., 2003).

Not performing oral care can cause some serious diseases and complications which would certainly lead to a rise in hospitalization time, caring costs and also mortality rate (Adib-Hajbaghery et al., 2011). Oral care not only has some short-term effects but this is able to prevent long-term complications specially halitosis, tooth decay, periodontal diseases, sinus infection as well as ventilator associated pneumonia (Ranjbar et al., 2011; Sole et al., 2003).

There are two methods for oral care, removing dental plaque and oral microorganisms including mechanical and pharmacological intervention. In healthy people, Mechanical clearance (using toothbrush) of teeth and gums plays an important role in prevention of producing biofilm and plaque. Brushing has been known as a necessary activity for oral hygiene promotion (Ames, 2011). Despite of being awareness of nurses concerning role of brushing in deleting dental plaque and also being comfortable to use, they are not inclined to implement this for their patients and it is often reported that only 41 percent of nurses utilize toothbrush for oral care in the patients.

Although it is evident that using toothbrush is more effective than swap in caring of patients’ oral hospitalizing in intensive care unit, many nurses prefer swap to toothbrush. A study indicated that toothbrush was used by just 34 percent of nurses whereas, 66% of them applied some Gauze parts for oral care.

There is reported that toothbrush and toothpaste are implied in patients without tracheal tube more than those supported with this (Dekeyser et al., 2009). Grifth and her colleagues suggested using a small and soft toothbrush in patients hospitalized in intensive care unit as, this kind helps to brush posterior parts of mouth and also tongue, gum and teeth (Cindy et al., 2004).

Medicinal intervention includes removing plaque and microorganisms by means of bactericidal agents. Some researchers have cleared that local antibiotics are effectiveness on washing and oral care; however this method was not performed because of producing microbial resistance (Adib-Hajbaghery et al., 2011). Some different solutions like chlorhexidine, sodium bicarbonate, hydrogen peroxide, sodium chloride, water, dilute Povidone-iodine, lemon juice and glycerin have been introduced to use as a mouth rinse. These days, chlorhexidine solution is the most favorable way for oral care (Berry & Davidson, 2006). Though this is amply documented that chlorhexidine is influential in declining negative bacterial colony in patients with open heart surgery, there are not found sufficient evidences for using this in other intensive care unit (Cindy et al., 2004). This is not recommended a specific instruction as a gold standard for oral care in intubated patients. Some advices have been offered by Center of disease prevention but they are often performed in different ways since, this is not approved scientifically (Feider et al., 2011). This is crucial that nurses working in the intensive care unit give priority to the oral care as an effective factor in patients’ quality of life.

Studies discus that common utilization of chlorhexidine should not been advised for all patients in intensive care unit. On the one hand, toothbrush and toothpaste are available and easy to use and on the other hand, chlorhexidine may create some side effects and resistance to the antibiotics. Enhance, this study aimed to compare the effectiveness of chlorhexidine solution with toothbrush on preventing oral lesions.

## 2. Methods

This clinical trial survey was carried out on patients hospitalizing in intensive care unit of shahid Beheshti hospital in Sabzevar, Iran. 30 patients were selected with target-based approach and divided into two groups through the permutation blocking method for oral care by means of toothbrush and toothpaste and using chlorhexidine and swab. In fact, this method guarantees that the number of subjects in two groups is equal leading to increasing tests power. In this way, some blocks were made including different permutations with 4 subjects (2 persons in toothbrush group and 2 persons in chlorhexidine). After this, the blocks were randomly made with number and selected flowed by a performance according to mutation of intrablocks.

In the first step, the aim of the study was explained to patient’s family to achieve their satisfaction for doing this experiment. Needed informati on was gathered from patient’s family via an interview with them. Oral hygiene was assessed by means of a modified version of Beck Oral Assessment Scale (BOAS) and MPS. The validity of these tools was approved by Safarabadi and Rezaie (Safarabady et al., 2013). The reliability of the questionnaire was determined by Cronbach’s alpha and internal consistency for this measure was 0.92.

BOAS examines oral hygiene in 5 parts as lips, gums, oral mucus, tongue, teeth and saliva measured on a four-point Likert. This scale totally ranging from 5 (without dysfunction) to 20 (strong dysfunction). MPS consist of two parts such as plaque and mucus which of them scoring up to 4 and obtaining fewer score shows the better oral health.

Inclusion criteria were: being intubated through the mouth over the study, aged between 18 to 65, not having severed injuries and sore in the mouth, history and signs of aspiration and coagulopathy, dentures and finally not suffering from hepatitis B and HIV. Exclusion criteria included transferring patient from intensive care unit to another unit, her or his death before completing the study, removal of tracheal tube, observation of clear aspiration, creation of dermal reaction, any limitation in performing technique and the possibility of aspiration.

The first intervention was done on patient who met the inclusion criteria at the admission time and then, every 8 hours. In order to do process, after washing hands by means of water and soap for 30 seconds and wearing disposable gloves, patient’s mouth was opened gently and its inside observed via flashlight. In the next step, patient’s mouth was examined through BOAS and MPS and recorded its features in a checklist. In the group receiving chlorhexidine solution before doing the technique, the head of bed would have raised up to 30 degree if there had not been any contraindication.

In the toothbrush group, a childish and soft toothbrush was utilized to brush all of the internal and external parts of teeth, gums, and the surfaces of tongue and palate with rotational and back to front movements, respectively. Afterwards, the mouth was cleared with amount of sterile distilled water by means of an applicator. In another group, mouth, tongue and teeth were washed using some cotton soaked in 2% chlorhexidine solution for 2 minutes based on the common method of unit. In both groups, these instructions were performed every 8 hours during 4 days. In order to prevent bias, we used two different parsons for examination and intervention. Finally, Data were analyzed by means of R software (version 3.0.2) and also Kolmogorov- Smirnov, multiple logistic regressions for correlated data, exact Fisher test.

## 3. Results

To compare of toothbrush with chlorhexidine solution in prevention of oral lesions based on multiple logistic regressions showed that the effects of the former significantly differ from the letter. As, patients in toothbrush group had a greater likelihood of having higher oral health levels than patients in another one (OR = 1.52). (Table 1).

**Table 1:** comparison between toothbrush and chlorhexidine solution in prevention of oral lesions based on multiple logistic regression for correlated data

Parameter	OR	S.E	p-value
**Age**	1.02654	0.0051	0.0001
**Group**	1.523484	0.1487	0.0046
**Sex**	1.48691	0.1550	0.0105
**Drugs**	1.594085	0.1573	0.0030

In addition, patients who were female and younger as one year had more likelihood of having healthier mouth (OR = 1.026 and 1.48). Besides, patients receiving Aminoglycosides were more likely to have the better oral health in comparison to patients were treated with Cephalosporin (OR = 1.48).

Moreover, there was no difference between two groups in preventing lesions in gums, lips and tongue (Table 2) while, this was significant with regards to the mucus (Table 5), plaque and teeth (Table 3). In the group of toothbrush subjects had stronger probability of having more suitable oral health. (OR = 2.75 and 3.94). In contrast, in the case of using chlorhexidine solution the patients were higher likely to have healthier saliva (OR = 2.046) (Table 4).

**Table 2:** Comparison between toothbrush and chlorhexidine solution in prevention of oral lesions in lips, gums and tongue based on multiple logistic regression for correlated data

Part of assess	Parameter	OR	S.E	p-value
**lips**	Group	0.9924288	0.3947	0.9846
**gums**	Group	1.59568	0.3959	0.2378
**tongue**	Group	1.632643	0.3870	0.2053

**Table 3:** Comparison between toothbrush and chlorhexidine solution in prevention of oral lesions in teeth and plug based on multiple logistic regressions for correlated data

Parameter	OR	S.E	p-value
**Age**	1.05211	0.0110	0.0001
**Group**	3.941652	0.3716	0.0002
**Sex**	1.6122	0.3863	0.2163
**Drugs**	1.7930	0.3741	0.1186

**Table 4:** Comparison between toothbrush and chlorhexidine solution in prevention of oral lesions in saliva based on multiple logistic regression for correlated data

Parameter	OR	S.E	p-value
**Age**	1.0104	0.0109	0.3415
**Group**	2.046232	0.3438	0.0373
**Sex**	1.1235	0.3846	0.7620
**Drugs**	1.3210	0.3247	0.1186

**Table 5:** Comparison between toothbrush and chlorhexidine solution in prevention of oral lesions in mucosa based on multiple logistic regression for correlated data

Parameter	OR	S.E	p-value
**Age**	1.0411	0.0142	0.0045
**Group**	2.753299	0.4137	0.0144
**Sex**	3.183878	0.4951	0.0193
**Drugs**	2.02	0.4676	0.1311

## 4. Discussion

The fundamental goal of oral care is to enhance oral health levels which would result in a decreased amount of oropharyngeal colony and dental plaques produced by bacterial infections and also aspiration of saliva including this colony (Feider et al., 2011). The present study was conducted to compare the preventive impact of toothbrush and chlorhexidine solution on oral lesions in patients hospitalized in intensive care unit. Obtained results revealed that both methods are helpful; however, in the group using toothbrush the speed of oral improvement was higher than another group. In a study done by AMS on 116 patients hospitalized in the intensive care unit, the effectiveness of a regular oral caring program compared with a routine caring program performed according to the protocol of unit. He resulted the regular care can be significantly more effective on improving oral health than routine care (Ames et al., 2011). Additionally, the findings of a systematic review study indicated that though mechanical ways such as toothbrush are at a higher priority than chlorhexidine solution to decline plaque colony, medicinal interventions like using mouth rinse solution have the stronger influence on oropharyngeal colony and ventilator-associated pneumonia (Fields, 2008). This study showed that using toothbrush is significantly associated to oral lesions in teeth and plaque. Based on results of the present study and others, it seems that dental plaque has been one of the most substantial indexes in evaluating oral hygiene. The dental plaque can be a location for accumulation of pathogenic microorganisms and also may play a marked role in creation of respiratory tract infection, for this reason toothbrush should be considered as a beneficial instrument for removing oral plaque. Time duration, the way of using and the type of toothbrush (having a long and flexible handle) can be effective in the process of plaque removal, as well. In regard to the oral saliva, chlorhexidine solution was more useful than toothbrush in maintaining health levels of saliva. In the komaris’ study, chlorhexidine solution showed the better results in improvement of oral saliva when this compared with normal saline (Kumari, 2013). Some factors like opening of mouth due to having tracheal intubation, not receiving fluids through the mouth, being diabetic and dehydration can cause Xerostomia. Because chlorhexidine solution has a liquid form, it is highly likely to lead to a drop in saliva concentration and this feature can be a reason for being more effective than toothbrush. Concerning oral mucus, findings cleared that this part is more affected by toothbrush than chlorhexidine solution. Crean illustrated that the toothbrush is the most effective means for clearing of mouth and removing dental plaque, gums and mucus (Kearns et al., 2009). There have been reported that dental plaque is as a result of colonization and growth of the various microorganisms on teeth surfaces, soft tissue and prosthodontics. If this is not removed, this would result in gingivitis over the less than 10 days. Gingivitis has been known as the first stage of periodontal disease and would certainly spread to all parts of the mouth if this is not treated. This finding is logical since, toothbrush acts stronger than chlorhexidine solution in plaque removal and prevents continuation of the disease. There are some positive points for using toothbrush instead of chlorhexidine. First, chlorhexidine is able to produce some complications and resistance to the antibiotics and so, this is not advised to use in all patients in intensive care unit. Secondly, utilizing toothbrush and toothpaste is really cost effective and easy to implement. As a result, this would be a reasonable idea to provide a toothbrush as a preventive strategy at the time of admission in the intensive care unit.

## 5. Limitations

One of the restrictions in this study was low sampling size owing to a few numbers of beds in this unit, being hospitalized for more than 2 months, transition to another unit or dying before completing questioners. This issue can limit generalization of results. Another limitation was time duration of intervention. Because of some restrictions concerning continued presence of the patients in the unit and also according to some studies in this field, we planned a four day intervention.

## 6. Conclusion

The present survey revealed that although toothbrush and chlorhexidine can prevent oral lesions, implementing toothbrush has greater effects on oral hygiene level.
